# Association of cooking oil and incident of frailty in older adults: a cohort study

**DOI:** 10.1186/s12877-024-05052-8

**Published:** 2024-05-13

**Authors:** Miao Dai, Huaping Xin, Weiwei Dai, Xiaohong Huang, Xiang Wang

**Affiliations:** 1 Department of Geriatrics, Jiujiang First People’s Hospital, Jiujiang, Jiangxi 332000 China; 2 Department of Geriatrics, Yichun People’s Hospital, Yichun, Jiangxi 330600 China; 3 Department of Dermatology, Jiujiang First People’s Hospital, Jiujiang, Jiangxi 332000 China; 4 Department of Cardiology, Jiujiang First People’s Hospital, Jiujiang, Jiangxi 332000 China

**Keywords:** Frailty, Vegetable oil, Animal fat oil, Older adult, Cohort study

## Abstract

**Background:**

Studies examining the potential association between cooking oil and frailty risk in older adults have produced conflicting outcomes. Therefore, our objective was to explore the relationship between cooking oil (vegetable and animal fat oils), changes in oil usage, and the risk of frailty in older adults.

**Methods:**

We included 4,838 participants aged ≥ 65 years without frailty (frailty index < 0.25) from the 2011 wave of the Chinese Longitudinal Healthy Longevity Survey. Follow-up occurred in the 2014 and 2018 waves. Cox proportional hazard models were utilized to estimate hazard ratios (HRs) and 95% confidence intervals (CIs) to examine the association between cooking oil and frailty. Additionally, we evaluated the effect of switching cooking oil on frailty during the follow-up period.

**Results:**

During a median follow-up of 3.0 (2.8–6.9) years, 1,348 individuals (27.9%) developed frailty. Compared to those using vegetable oil, users of animal fat oil had a lower risk of frailty (HR = 0.72, 95% CI: 0.61–0.85). Participants who switched from vegetable oil to animal fat oil, as well as those consistently using animal fat oil, had lower risks of frailty with HRs of 0.70 (0.52–0.95) and 0.63 (0.51–0.77) respectively, compared to those who consistently used vegetable oil. Conversely, individuals who switched from animal fat oil to vegetable oil experienced an increased risk of frailty (HR: 1.41, 95% CI: 1.01–1.97).

**Conclusions:**

The utilization of animal fat oil in cooking exhibited a reduced frailty risk among older adults. Conversely, transitioning from animal fat oil to vegetable oil may elevate the risk. These findings propose that substituting vegetable oil with animal fat oil in the diet may safeguard against frailty.

**Supplementary Information:**

The online version contains supplementary material available at 10.1186/s12877-024-05052-8.

## Background

Frailty, a prevalent geriatric syndrome characterized by diminished physiological capacity and heightened susceptibility to stressors [1], entails adverse health outcomes such as falls [2], disability [3], and mortality [4]. A systematic review revealed that, among individuals aged 50 years and above across 62 countries, the occurrence of prefrailty and frailty was determined to be 49% and 24%, respectively [5]. Notably, the prevalence of frailty showed an upward trend with advancing age, reflecting the impact of global population aging [5]. Among older individuals, frailty is a dynamic process characterized by transitions between different frailty states over time, indicating a substantial potential for the prevention and management of frailty [6]. Therefore, the identification of modifiable risk factors associated with frailty assumes paramount importance in mitigating or postponing the onset of this condition.

Diet is a modifiable risk factor for frailty. A growing body of evidence suggests that dietary patterns rich in fruits, vegetables, whole grains, and lean protein sources are associated with a lower risk of frailty in older adults [7, 8]. Vegetable oils and animal fat oils are two of the most common sources of fat in the Chinese diet. Vegetable oils, such as soybean oil, corn oil, and canola oil, are rich in unsaturated fatty acids (UFAs) [9, 10]. In contrast, animal fat oils, such as lard and tallow, are rich in saturated fatty acids (SFAs) [11]. Several studies have investigated the association between dietary fat intake and frailty among older adults, but the results have been inconsistent. For example, one cross-sectional study reported that a higher intake of SFAs was associated with an increased risk of frailty in American adults aged 50 years or older [12]. Conversely, a prospective cohort of 1,822 community-dwelling individuals aged 60 and older in Spain found that UFAs were linked to lower frailty risk, while it has found no significant association between SFAs and the risk of frailty [13]. Little is currently known about whether the results of previous studies can be generalized to older adults in China, nor is it clear whether there is an association between changes in the type of cooking oil during the follow-up period and frailty. Given the high prevalence of frailty, prospective studies are needed to clarify this critical issue.

Therefore, this study aimed to examine the association between cooking oil including vegetable oil and animal fat oil, transitions in cooking oil, and frailty in older adults using data from the Chinese Longitudinal Healthy Longevity Survey (CLHLS).

## Methods

### Study design and participants

The CLHLS is a prospective cohort study designed to explore determinants of healthy aging and longevity among the older population in China. The study spans 22 out of 31 provinces in China and encompasses a comprehensive representation of the country’s demography, covering around 85% of the Chinese populace. The survey has been ongoing since 1998 and the project has conducted follow-up surveys in 2000, 2002, 2005, 2008–2009, 2011–2012, 2014, and 2017–2018. To counteract attrition caused by mortality and loss of follow-up, new participants have been enrolled at every follow-up, and surviving participants have been re-interviewed. The survey employs structured questionnaires administered by trained interviewers at participants’ homes. Detailed information about CLHLS is available elsewhere [14].

**Fig. 1:**
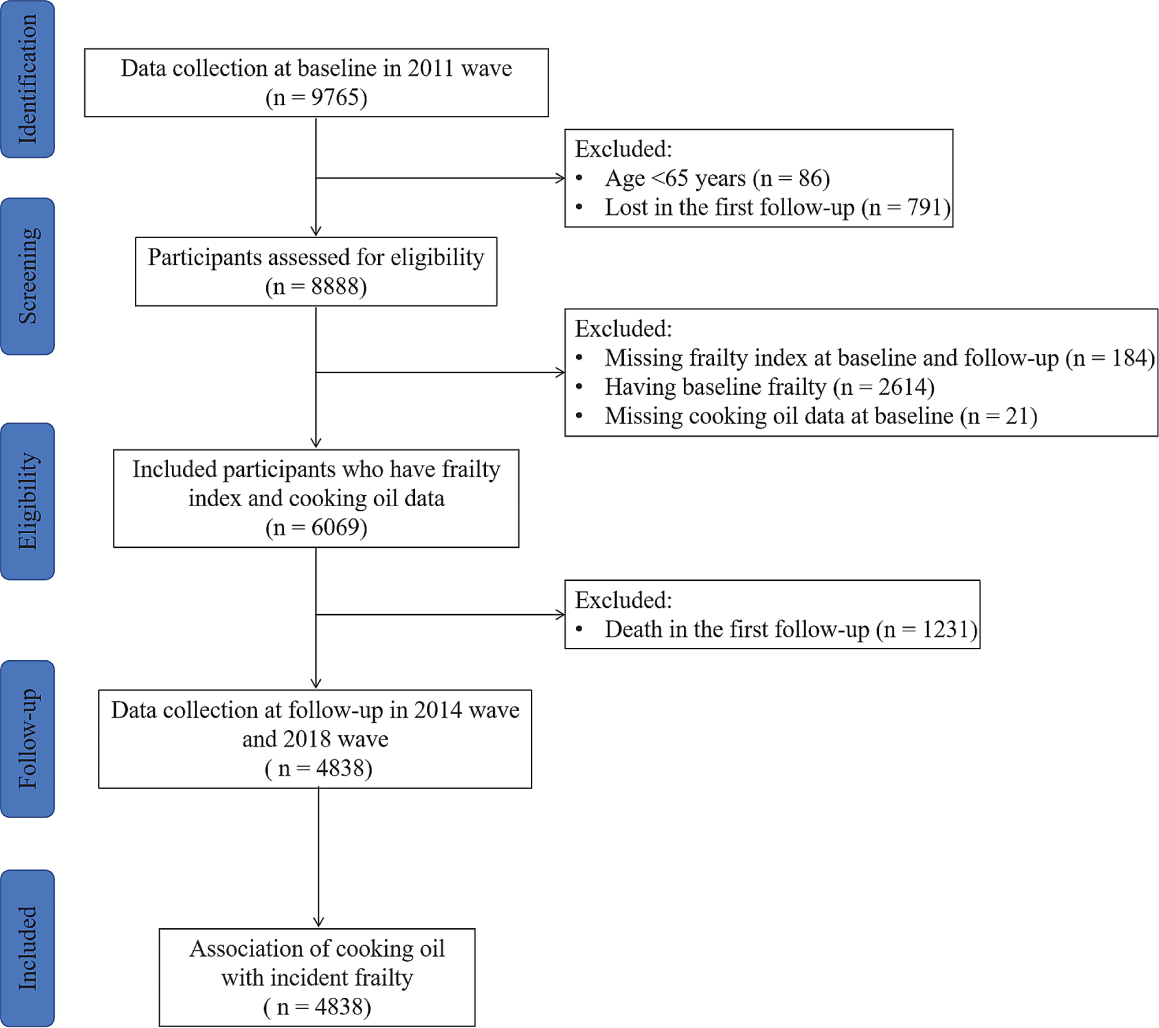
Flowchart of the inclusion of study participants

To reflect the latest cooking oil use and frailty situation of Chinese older adults in CLHLS, the baseline data for this study were obtained from the 2011 wave of the CLHLS. Following the inclusion of Chengmai City in Hainan Province, this wave was expanded to cover 23 provinces (Supplementary Fig. 1). Subsequently, follow-up surveys were conducted in 2014 and 2017/2018. A total of 9,765 older adults were initially included in the study. Participants who were younger than 65 years old (*n* = 86), lost to follow-up in the 2014 wave (*n* = 791), had missing data on frailty index (FI) at baseline and follow-up (*n* = 184), frailty at baseline (*n* = 2,614), had missing data on cooking oil at baseline (*n* = 21), and death during follow-up in the 2014 wave (*n* = 1,231) were excluded. Finally, a total of 4,838 participants were included to analyze the association of cooking oil with incident frailty in older adults. To investigate the association between longitudinal changes in cooking oil over time and the risk of frailty, we additionally excluded participants with missing data on cooking oil in the 2014 wave (*n* = 34). Figure 1 provides a detailed description of the inclusion and exclusion process.

### Cooking oil assessment

To determine the type of cooking oil used, participants or a close relative of the interviewee completed a questionnaire that asked, “What kind of oil do you mainly use for cooking?” We compared the primary use of vegetable oil (vegetable oil or sesame oil) with the primary use of animal fat oil (lard or other animal fat). Additionally, we examined self-reported switching between vegetable oil and animal fat oil, which involved participants who initially used one type of oil but switched to the other during the follow-up period in the 2014 wave.

### Frailty assessment

Frailty status was assessed using the FI, a widely used tool that measures health deficits. To construct the FI, 39 items were used following a standardized procedure (Supplementary Table 1). These items evaluated various aspects of health, including self-rated and interviewer-rated health, cognitive function, psychological characteristics, disability in daily activities, hearing abilities, vision abilities, functional limitations, and chronic diseases. Some health deficits were binary-coded, with “1” indicating the presence of deficits and “0” indicating their absence. An ordered classification variable was also utilized, with scores ranging from 0 to (1) Respondents who were bedridden or had more than one serious illness within the past two years were assigned a score of (2) The FI was computed by adding up the scores of all variables and dividing them by the total number of variables considered. The resulting score ranged from 0 to 1. Frailty was categorized as non-frailty (FI < 0.15), pre-frail (0.15–0.25), and frailty (≥ 0.25) [15, 16].

### Covariates assessment

We included baseline sociodemographic characteristics, lifestyle factors, and health status in our model. These particular covariates were chosen in advance as potential confounders, informed by existing literature [17–24]. The sociodemographic characteristics section included age (continuous), sex (male or female), education (0 years, 1–6 years, or ≥ 6 years), residence (rural area or urban area), living with family members (yes or no), economic status (independence or dependence), and marital status (married or others [divorced, widowed, or never married]). The lifestyle section includes smoking (never, current or former), alcohol consumption (never, current or former), regular exercise (never, current or former), frequency of vegetable intake (daily, quite often, occasionally, or rarely or none), frequency of fruit intake (daily, quite often, occasionally, or rarely or none), frequency of meat intake (daily, weekly, monthly, occasionally, or rarely or none), frequency of fish intake (daily, weekly, monthly, occasionally, or rarely or none). Health status consisted of body mass index (BMI), frailty status (non-frailty or prefrailty), number of natural teeth (20+, 10–19, 1–9, or 0), and denture use (yes or no). BMI was calculated as weight (in kg) divided by height (in meters squared) and categorized as underweight (BMI < 18.5 kg/m^2^), normal weight (18.5–24 kg/m^2^), overweight (24–28 kg/m^2^), or obese (≥ 28 kg/m^2^) [25].

### Statistical analysis

We presented baseline characteristics as means and standard deviations (SDs) for continuous variables and as percentages for categorical variables. We performed multiple imputations based on 5 replications and the chained equation approach method to address missing data [26] (ranging from 0.02 to 1.0%) (Supplementary Table 2).

The follow-up period concluded on the date when incident frailty occurred, or at the end of the study, whichever came first. We used Cox proportional hazards regression models to estimate hazard ratios (HRs) and 95% confidence intervals (CIs) for the association between cooking oil (vegetable oil and animal fat oil) and the risk of frailty. The proportional hazards assumption was tested and found to be met. We first examined the unadjusted association between cooking oil and frailty. We then adjusted for age and sex in model 1, and subsequently further adjusted for residence, education, living arrangement, economic status, marital status, smoking status, drinking status, regular exercise, the number of natural teeth, denture use, BMI, baseline frailty status, frequency of fruit intake, frequency of vegetable intake, frequency of meat intake, frequency of fish intake in model 2. The crude incidence rate per 100 person-years of incident frailty across categories of cooking oil was estimated. We tested interaction terms of potential covariates (age, sex, residence, education, living arrangement, economic status, marital status, smoking status, drinking status, regular exercise, the number of natural teeth, denture use, BMI, and baseline frailty status) with cooking oil on incident frailty and performed subgroup analyses, and *P*-value for interaction were adjusted using Bonferroni corrections. In addition, to mitigate the potential influence of competing risk bias on our findings, we performed a competing risk model analysis to assess the risk of frailty, considering death as a competing event. The calculations of cumulative incidence in the competing risk analysis were carried out using the “cmprsk” package in R. We also assessed the risk of frailty among participants who reported switching cooking oil during the follow-up period compared to those who did not.

We conducted several sensitivity analyses to ensure the reliability of our findings. Firstly, we utilized propensity score matching (PSM) to address the influence of confounding variables by balancing the baseline characteristics between the observation group (animal fat oil) and the control group (vegetable oil), thereby replicating the expected effects of randomization and reducing confounding [27]. We assessed the balance between the groups using the standardized mean difference (SMD), aiming for an SMD of less than 0.1 to indicate a balanced distribution of covariates [28]. Secondly, we performed the analysis only on complete cases to evaluate the potential impact of the multiple imputation approach. Thirdly, we excluded cases of frailty that occurred within the first two years of follow-up to minimize potential reverse causality. Lastly, we accounted for additional factors such as sleep time, sleep quality (very good, good, so so, bad, very bad), frequency of egg intake (daily, quite often, occasionally, or rarely or none), frequency of bean intake (daily, quite often, occasionally, or rarely or none), frequency of milk intake (daily, weekly, monthly, occasionally, or rarely or none), and amount of staple food per day (Liang) in our analysis.

The statistical software R (version 4.1.3, R Foundation for Statistical Computing) was utilized for all statistical analyses, with statistical significance set at a two-tailed *P* value < 0.05.

## Results

### Basic characteristics of participants

Among the 4838 participants, 2257 (48.7%) were pre-frail, 2481 (51.3%) were non-frail, and 49.7% were female, with a mean (SD) age of 80.8 (9.6) years. With a median follow-up period of 3.0 years (interquartile range: 2.8 to 6.9, 21876.8 person-years), 1348 (27.9%) incident frailty was identified. Table 1 shows the characteristics of the participants by cooking oil. The participants who reported using animal fat oil tended to be older, reside in rural areas, be unmarried, have lower levels of education, and be economically dependent, as compared to those who reported using vegetable oil. Additionally, animal fat oil users were more likely to not smoke or engage in regular exercise, be non-frail, have a normal BMI, have few natural teeth, not use dentures, have an insufficient frequency of fruit, vegetable, and fish intake, and have a sufficient frequency of meat intake.

The characteristics of participants who switched cooking oil types during follow-up are presented in Supplementary Table 3. Compared to participants who consistently used vegetable oil, those who switched from vegetable oil to animal fat oil tended to be older, reside in rural areas, be unmarried, have lower levels of education, and be economically dependent. Furthermore, individuals using animal fat oil were more likely to abstain from smoking or engaging in regular exercise, have a normal BMI, possess fewer natural teeth, use dentures, have insufficient frequencies of fruit, vegetable, and fish intake, and have a sufficient frequency of meat intake.

**Table 1:** Baseline characteristics of participants according to types of cooking oil

Characteristics	All participants (*n* = 4838)	Vegetable oil (*n* = 4140)	Animal fat oil (*n* = 698)	*P* value ^a^
Age (year), mean (SD)	80.76 (9.58)	80.46 (9.54)	82.59 (9.59)	< 0.001
Female, no. (%)	2405 (49.7)	2049 (49.5)	356 (51.0)	0.49
Urban area, no. (%)	2247 (46.4)	2046 (49.4)	201 (28.8)	< 0.001
Married, no. (%)	2440 (50.4)	2126 (51.4)	314 (45.0)	0.02
Living with family, no. (%)	3829 (79.1)	3295 (79.6)	534 (76.5)	0.07
Education (year), no. (%)				< 0.001
0	2439 (50.4)	2046 (49.4)	393 (56.3)	
1–6	1750 (36.2)	1504 (36.3)	246 (35.2)	
> 6	649 (13.4)	590 (14.3)	59 (8.5)	
Economic independence, no. (%)	1672 (34.6)	1543 (37.3)	129 (18.5)	< 0.001
Smoking status, no. (%)				0.003
Never	3018 (62.4)	2553 (61.7)	465 (66.6)	
Current	1065 (22.0)	911 (22.0)	154 (22.1)	
Former	755 (15.6)	676 (16.3)	79 (11.3)	
Drinking status, no. (%)				0.62
Never	3153 (65.2)	2709 (65.4)	444 (63.6)	
Current	1009 (20.9)	855 (20.7)	154 (22.1)	
Former	676 (14.0)	576 (13.9)	100 (14.3)	
Regular exercise, no. (%)				< 0.001
Never	2393 (49.5)	1994 (48.2)	399 (57.2)	
Current	2051 (42.4)	1809 (43.7)	242 (34.7)	
Former	394 (8.1)	337 (8.1)	57 (8.2)	
Frailty status				0.04
Non-frailty	3764 (77.8)	3200 (77.3)	564 (80.8)	
Pre-frailty	1074 (22.2)	940 (22.7)	134 (19.2)	
BMI (kg/m^2^), no. (%)				< 0.001
Underweight (< 18.5)	2737 (56.6)	2354 (56.9)	383 (54.9)	
Normal (18.5–24)	918 (19.0)	696 (16.8)	222 (31.8)	
Overweight (24–28)	904 (18.7)	832 (20.1)	72 (10.3)	
Obese (≥ 28)	279 (5.8)	258 (6.2)	21 (3.0)	
Natural tooth number, no. (%)				< 0.001
20+	1399 (28.9)	1240 (30.0)	159 (22.8)	
10–19	903 (18.7)	782 (18.9)	121 (17.3)	
1–9	1290 (26.7)	1080 (26.1)	210 (30.1)	
0	1246 (25.8)	1038 (25.1)	208 (29.8)	
Denture use, no. (%)	1850 (38.2)	1638 (39.6)	212 (30.4)	< 0.001
Frequency of fruit intake, no. (%)				< 0.001
Daily	658 (13.6)	619 (15.0)	39 (5.6)	
Quite often	1225 (25.3)	1100 (26.6)	125 (17.9)	
Occasionally	1737 (35.9)	1436 (34.7)	301 (43.1)	
Rarely or none	1218 (25.2)	985 (23.8)	233 (33.4)	
Frequency of vegetable intake, no. (%)				< 0.001
Daily	2959 (61.2)	2571 (62.1)	388 (55.6)	
Quite often	1486 (30.7)	1272 (30.7)	214 (30.7)	
Occasionally	295 (6.1)	237 (5.7)	58 (8.3)	
Rarely or none	98 (2.0)	60 (1.4)	38 (5.4)	
Frequency of meat intake, no. (%)				< 0.001
Daily	1514 (31.3)	1204 (29.1)	310 (44.4)	< 0.001
Weekly	2098 (43.4)	1836 (44.3)	262 (37.5)	
Monthly	575 (11.9)	506 (12.2)	69 (9.9)	
Occasionally	316 (6.5)	286 (6.9)	30 (4.3)	
Rarely or none	335 (6.9)	308 (7.4)	27 (3.9)	
Frequency of fish intake, no. (%)				< 0.001
Daily	378 (7.8)	347 (8.4)	31 (4.4)	
Weekly	1823 (37.7)	1630 (39.4)	193 (27.7)	
Monthly	1065 (22.0)	882 (21.3)	183 (26.2)	
Occasionally	676 (14.0)	560 (13.5)	116 (16.6)	
Rarely or none	896 (18.5)	721 (17.4)	175 (25.1)	

*SD* standard deviation; *BMI* Body mass index

*Notes*: Values are presented as number (%) or mean ± SD. ^a^ Differences in characteristics were compared using the χ^2^ test for categorical variables and the t-test for continuous variables

After performing the PSM, a total of 1350 participants were included in the analysis. The SMDs of the covariates were all below 0.1, suggesting that the groups resulting from PSM were well-balanced and exhibited no significant confounding effects (Supplementary Fig. 2). Following PSM, the baseline variables between the vegetable oil and animal fat oil groups were effectively balanced (Supplementary Table 4).

### Association of cooking oil with incident frailty

After adjusting for potential confounding factors, participants who consumed animal fat oil had a significantly lower risk of frailty compared to those who consumed vegetable oil (IR per 100 person-years: 5.7 versus 6.2; HR = 0.72, 95% CI = 0.61–0.85) (Table 2). The competing risk model analysis revealed a similar pattern (Supplementary Table 5).

**Table 2:** Hazard ratios for incident frailty according to cooking oil categories

Cooking oil	No. Of events/total	Incidence rate ^a^	Unadjusted Model	Model 1	Model 2
			HR (95% CI)	HR (95% CI)	HR (95% CI)
Vegetable oil	1163 (4140)	6.2	Reference	Reference	Reference
Animal fat oil	185 (698)	5.7	0.84 (0.72–0.98)	0.69 (0.59–0.80)	0.72 (0.61–0.85)

*HR* hazard ratio; *CI* confidence interval

Notes: ^a^ Incidence rates per 100 person-years

Model 1: adjusted for age and sex

Model 2: further adjusted for residence, education, living arrangement, economic status, marital status, smoking status, drinking status, regular exercise, the number of natural teeth, denture use, body mass index, baseline frailty status, frequency of fruit intake, frequency of vegetable intake, frequency of meat intake, and frequency of fish intake

### Association of transitions in cooking oil types and frailty

The risk of frailty was lower in persistent animal fat oil users or those who reported switching from vegetable oil to animal fat oil than in persistent vegetable oil users during follow-up, the HRs were 0.63 (0.51–0.77) and 0.70 (0.52–0.95), respectively, but no significant association between switching from animal fat oil to vegetable oil and frailty was found (Table 3). Compared with persistent vegetable oil users, participants who reported switching from vegetable oil to animal fat oil for cooking had a lower risk of frailty (IR per 100 person-years: 5.5 versus 6.3; HR: 0.69, 95% CI: 0.51–0.95) (Table 3). In addition, compared with persistent animal fat oil users, participants who reported switching from animal fat oil to vegetable oil for cooking had a noticeably higher risk of frailty (IR per 100 person-years: 7.0 versus 5.2; HR: 1.41, 95% CI: 1.01–1.97) (Table 3).

**Table 3:** Association between switching cooking oil and incident frailty

Cooking oil	No. Of events /total	Incidence rate ^a^	Unadjusted Model	Model 1	Model 2
			HR (95% CI)	HR (95% CI)	HR (95% CI)
Always vegetable oil	1109 (3933)	6.3	Reference	Reference	Reference
Vegetable oil to animal fat oil	45 (176)	5.5	0.76 (0.56–1.03)	0.66 (0.49–0.89)	0.70 (0.52–0.95)
Animal fat oil to vegetable oil	68 (212)	7.0	1.01 (0.79–1.29)	0.80 (0.62–1.02)	0.87 (0.68–1.12)
Always animal fat oil	116 (483)	5.2	0.75 (0.62–0.91)	0.61 (0.51–0.74)	0.63 (0.51–0.77)
Switch from vegetable oil to animal fat oil					
Always vegetable oil	1109 (3933)	6.3	Reference	Reference	Reference
Vegetable oil to animal fat oil	45 (176)	5.5	0.75 (0.56–1.02)	0.65 (0.48–0.88)	0.69 (0.51–0.95)
Switch from animal fat oil to vegetable oil					
Always animal fat oil	116 (483)	5.2	Reference	Reference	Reference
Animal fat oil to vegetable oil	68 (212)	7.0	1.31 (0.97–1.78)	1.26 (0.93–1.71)	1.41 (1.01–1.97)

*HR* hazard ratio; *CI* confidence interval

*Notes*: ^a^ Incidence rates per 100 person-years

Model 1: adjusted for age and sex

Model 2: further adjusted for residence, education, living arrangement, economic status, marital status, smoking status, drinking status, regular exercise, the number of natural teeth, denture use, body mass index, baseline frailty status, frequency of fruit intake, frequency of vegetable intake, frequency of meat intake, and frequency of fish intake

### Subgroup analyses

**Fig. 2:**
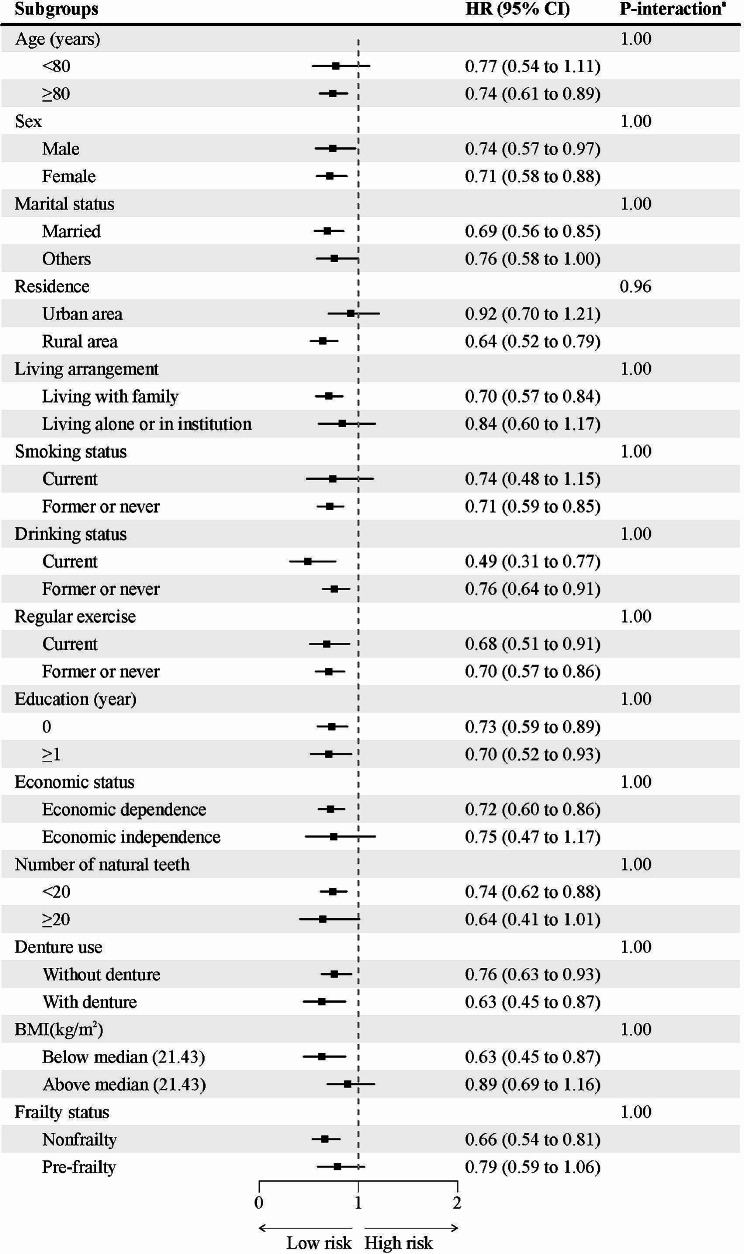
Association of cooking oil with incident frailty stratified by participant characteristics *HR* hazard ratio; *CI* confidence interval; *BMI* body mass index *Notes*: ^a^ Adjusted *P* value was shown with Bonferroni corrections Each stratification controlled for all factors (age, sex, residence, education, economic status, marital status, living arrangement, smoking status, drinking status, regular exercise, sleep time, the number of natural teeth, denture use, body mass index, frailty status, frequency of fruit intake, frequency of vegetable intake, frequency of meat intake, and frequency of fish intake.) except the stratification factor itself

In subgroup analyses, none of the included variables, including age, sex, marital status, residence, living arrangement, smoking status, drinking status, regular exercise, education, economic status, the number of natural teeth, denture use, BMI, and baseline frailty status, significantly modified the association between cooking oil and incident frailty (Fig. 2).

### Sensitivity analyses

To ensure the robustness of our study findings, we performed a series of sensitivity analyses.

The outcomes obtained from the PSM were consistent with our primary analysis (Supplementary Table 6). Moreover, the fully adjusted model, which was limited to participants with complete data, or excluded those who developed frailty during the second year of follow-up, or additionally adjusted for sleep time, sleep quality, frequency of egg intake, frequency of bean intake and frequency of milk intake, and amount of staple food per day, displayed consistent results with our initially adjusted and imputed model (Supplementary Table 6).

## Discussion

This prospective cohort study examined the association between cooking oil, transitions in cooking oil, and frailty among Chinese older adults. We found that older adults who consumed animal fat oil at baseline or persistent consumed animal fat oil during the follow-up had a significantly decreased risk of frailty compared with those who consumed vegetable oil. Conversely, transitioning from animal fat oil to vegetable oil during the follow-up period elevated the risk of frailty. The present study adds to the existing literature by providing evidence for the potential protective effect of animal fat oil on frailty risk in older adults.

The selection of oils and fats in cooking varies significantly across culinary traditions and geographical regions. In Europe and the United States, common animal fats like butter are prevalent, while vegetable fats such as olive oil are extensively used. Conversely, Chinese cuisine predominantly relies on lard as the primary animal fat oil, alongside a variety of vegetable oils like soybean oil, corn oil, canola oil, and sesame oil, among others. Stir-frying, deep-frying, and steaming are prevalent cooking methods in Chinese cuisine. Animal fat oils and vegetable oils are employed accordingly, with animal fat oils preferred for deep-frying due to their high smoke point and ability to endure high temperatures without imparting off-flavors. Sesame oil is often added towards the end of cooking or used as a flavoring agent in marinades and dressings to preserve its delicate flavor. The quantity of fats and oils utilized in Chinese cooking varies depending on the dish and regional culinary preferences. Stir-frying typically necessitates a moderate amount of oil to prevent sticking and evenly coat ingredients, whereas deep-frying may require larger quantities of oil. Chinese cooking often emphasizes quick cooking techniques at high temperatures, necessitating oils and fats with high smoke points to prevent burning and maintain food quality. Additionally, the choice of oil can influence the texture and mouthfeel of dishes, with animal fat oils lending a distinct richness and vegetable oils offering a lighter, cleaner taste.

However, the fatty acid composition found in vegetable and animal oils exhibits significant disparities. Abundant in UFAs, particularly monounsaturated and polyunsaturated fatty acids such as linoleic acid and linolenic acid, vegetable oils contrast with animal oils, which feature higher levels of SFAs like stearic acid and palmitic acid [29]. Furthermore, vegetable oils such as soybean oil, corn oil, and canola oil contain notable amounts of vitamin E, tocopherols, phytosterols, and omega-3 fatty acids, which confer antioxidant properties, thereby contributing to their nutritional value [30–32]. Sesame oil, often utilized as a flavor enhancer rather than a primary cooking oil, is rich in monounsaturated and polyunsaturated fats. Additionally, it serves as a good source of antioxidants such as sesamol and sesaminol, imparting a distinct nutty flavor to dishes [33]. Conversely, animal fat oils are rich in vitamin A and vitamin D, though they contain a lower proportion of fat compared to butter. The proportions and types of these fatty acids exert distinct effects on human health.

Although vegetable oils are generally perceived as healthier due to their reduced saturated fat content, some studies have raised doubts regarding the superiority of vegetable fats when compared to animal fats [34–36]. For example, Ramsden CE et al. demonstrated that replacing SFA with vegetable oils rich in linoleic acid can effectively lower serum cholesterol levels, but these interventions did not reduce the risk of coronary heart disease or overall mortality [36]. Our study revealed a significant association between the utilization of animal fat oils as a cooking oil and a reduced risk of frailty among older adults in China. However, our findings contradict previous studies that have shown an association between animal fat oil and a higher risk of frailty or have found no significant association between the two. For example, in one cross-sectional study, which included 4,062 participants aged 50 years and above, a higher percentage of SFA intake was found to be associated with a higher risk of frailty, even after considering the degree of nutritional deficits [12]. Sandoval-Insausti et al. discovered a negative correlation between the consumption of monounsaturated fatty acids and the risk of frailty in older adults during an average 3.5-year follow-up period [13]. In contrast, their findings indicated that the intake of SAF did not show a significant association with the risk of frailty. Potential explanations for the observed variations in the association between cooking oil and frailty among the study participants can be attributed to disparities in age, ethnicity, cultural ecology, lifestyle, or geographic location. We recruited Chinese older participants characterized by significantly advanced age (mean age 80 years) and an almost equal distribution of both genders. It is noteworthy that the association between the consumption of cooking oil and the state of frailty might be susceptible to the influence of a diverse array of distinct living practices and dietary patterns linked to variations in cooking techniques and levels of physical activity. Furthermore, our research distinguishes itself from previous investigations that were confined to singular geographical settings or restricted to small areas. In contrast, our study encompasses a substantial expanse, encompassing 23 research sites across 23 provinces in mainland China.

In our competitive risk model analysis, we noted a similar trend, revealing that individuals who consumed animal fat oils exhibited a notably reduced risk of frailty compared to those who consumed vegetable oils. However, the association between oil consumption and frailty risk was diluted when accounting for competing events. The attenuation towards the null in competing risk findings suggests that the association we observed may be influenced by other competing events or risks that prevent the occurrence of frailty, thereby influencing the magnitude and significance of the association. This attenuation underscores the complexity of the relationship between dietary factors and frailty, which may be modulated by various competing factors such as other health conditions, lifestyle factors, or demographic variables.

Additionally, our investigation further reveals that among older individuals, the transition from vegetable oil to animal oil as the primary cooking oil was correlated with a notable reduction in the vulnerability to frailty, as opposed to the continued use of vegetable oil. Conversely, the transition from animal to vegetable oil demonstrated the opposing effect on frailty. Given the paucity of extant literature, establishing a direct comparison between the correlation of frailty and cooking oil transformation in geriatric populations and prior investigations poses a challenge. Overall, as a precautionary measure, it is imperative to exercise caution regarding the frailty of older adults when considering the switch from animal oils to vegetable oils, given the potential associated health risks.

The potential mechanisms underlying the association between animal fat oil consumption and frailty are not fully understood, but several hypotheses have been proposed. Animal fat oil contains a higher proportion of saturated fats, which are more resistant to oxidative damage during cooking. The stability of animal fat oil at high temperatures may preserve its nutritional properties and reduce the formation of harmful byproducts, such as acrolein, hydroxynonenal, and trans-fatty acids [37, 38]. Moreover, animal fat oils are known for their rich flavor and ability to enhance the taste of dishes. This may contribute to improved appetite and nutrient intake among older adults, leading to better overall nutritional status and potentially reducing frailty risk. Conversely, vegetable oils, particularly those rich in ω-6 fatty acids, have been linked to heightened inflammation and oxidative stress [39, 40], which may contribute to age-related diseases such as frailty. Studies have indicated that the ω-6 polyunsaturated fatty acid-rich vegetable oil-derived compound “hydroxynonenal” could be the primary factor responsible for cellular degeneration and apoptosis, thereby facilitating the development of metabolic-related diseases [38, 41, 42]. One of the factors contributing to frailty is energy and protein undernutrition (PEM). The demographic characteristics associated with the consumption of animal fat oil, including older age, rural residence, lower education levels, and economic dependence, may indeed suggest access to a more traditional or natural diet rich in sources of animal fats and proteins. Individuals in these demographics may have greater access to home-raised animals or locally sourced meats, which can contribute to a higher intake of essential nutrients crucial for preventing PEM. Moreover, the observation that animal fat oil users are more likely to refrain from smoking, engage in regular exercise, and maintain a normal BMI indicates the possibility of leading healthier lifestyles overall. This healthier lifestyle may include a more balanced diet with sufficient amounts of protein and energy, thus potentially helping to prevent PEM. Additionally, while it is noted that individuals consuming animal fat oils tend to have insufficient intake of fruits, vegetables, and fish, their adequate intake of meat is noteworthy. Despite the concerns about overall nutritional adequacy due to the lack of fruits and vegetables, a sufficient intake of meat can provide essential proteins and energy, which are vital for preventing PEM. Furthermore, the observation that individuals consuming animal fat oils are more likely to have few natural teeth and not use dentures may indicate a preference for softer meats, which still provide essential nutrients necessary for preventing PEM. This preference for softer meats may also suggest a cultural or dietary pattern that includes a higher intake of animal-derived proteins and fats, contributing to the prevention of PEM. Overall, while our study highlights the potential benefits of utilizing animal fat oil in cooking for reducing frailty risk among older adults, further research is warranted to elucidate the specific dietary patterns and mechanisms underlying the observed associations with PEM prevention. Nevertheless, these hypotheses necessitate further empirical scrutiny utilizing specific datasets to authenticate the findings and unravel the underlying mechanisms at play.

### Strengths and limitations

The strengths of this study include the use of a validated measure of frailty, the large sample size, and the prospective design. We also assessed the association between longitudinal changes in cooking oil types over time and frailty. However, there are several limitations to consider. First, the study was observational in nature, and causality cannot be inferred. Second, dietary intake was self-reported and subject to recall bias. Third, we did not measure the types and amounts of specific fatty acids in the animal fat oil and vegetable oil consumed by the participants. Exploring the specific types of animal fat oil, preparation techniques, and dietary patterns associated with the observed protective effect would yield valuable insights for tailored dietary interventions. Fourth, we cannot exclude the possibility of residual confounding, despite our efforts to control for potential confounders. Finally, our study was conducted in China, and the results may not be generalizable to other populations.

## Conclusions

In conclusion, our cohort study suggests that animal fat oil consumption is associated with a significantly decreased risk of frailty among older adults. This finding challenges previous assumptions regarding the potential health effects of animal fat oil intake and highlights the importance of considering individual dietary patterns in the context of frailty prevention. Further research, including randomized controlled trials and mechanistic studies, is warranted to elucidate the underlying biological mechanisms and confirm these observations. Nonetheless, these findings hold promising implications for public health strategies aiming to reduce the burden of frailty and improve the well-being of older adults.

### Electronic supplementary material

Below is the link to the electronic supplementary material.

Supplementary Material 1

## Data Availability

Data are from the Chinese Longitudinal Healthy Longevity Survey, which is a public, open-access repository (https://opendata.pku.edu.cn/dataverse/CHADS).

## Abbreviations

FI: frailty index
CLHLS: Chinese Longitudinal Healthy Longevity Survey
UFA: unsaturated fatty acid
SFA: saturated fatty acid
BMI: body mass index
SD: standard deviation
PSM: propensity score matching
SMD: standardized mean difference
HR: hazard ratio
CI: confidence interval
