# Supplementary material for: Association of cooking oil and incident of frailty in older adults: a cohort study
Source: BMC Geriatr. 2024 May 13;24:424. doi: 10.1186/s12877-024-05052-8 (PMC11092255; doi:10.1186/s12877-024-05052-8)
Supplement: Supplementary file 1 — Supplementary Material 1 [file 12877_2024_5052_MOESM1_ESM.docx]

**Supplementary Table 1**

Health variables and cut-points for the frailty index

| NO. | Items | Cut point |
| --- | --- | --- |
| 1 | Self-rated quality of life | Very bad = 1, Bad= 0.75, So so = 0.5, Good = 0.25, Very good = 0 |
| 2 | Health worsened in the past year | Much worse = 1, A little worse = 0.75, No change = 0.5, A little better = 0.25, Much better = 0 |
| 3 | Look on the bright side of things | Never = 1, seldom = 0.75, sometimes = 0.5, often = 0.25, always = 0 |
| 4 | Feel fearful or anxious | Always = 1, Often =0.75, Sometimes = 0.5, Seldom = 0.25, Never = 0 |
| 5 | Feel lonely and isolated | Always = 1, Often =0.75, Sometimes = 0.5, Seldom = 0.25, Never = 0 |
| 6 | Feel useless with age | Always = 1, Often =0.75, Sometimes = 0.5, Seldom = 0.25, Never = 0 |
| 7 | Cognitively impaired (based on the Mini-Mental State Examination) | ≤10 = 1, 11-17 = 0.75, 18-20 = 0.5, 21-24 = 0.25, ≥25 = 0 |
| 8 | ADLs: Needs assistance bathing | More than one part assistance = 1, Partial assistance = 0.5, Without assistance = 0 |
| 9 | ADLs: Needs assistance dressing | Assistance in getting clothes and getting dressed = 1, Need assistance for trying shoes = 0.5, Without assistance = 0 |
| 10 | ADLs: Needs assistance toileting | Don't use toilet = 1, Assistance in cleaning or arranging clothes = 0.5, Without assistance = 0 |
| 11 | ADLs: Needs assistance in indoor transferring | Bedridden = 1, With assistance = 0.5, Without assistance = 0 |
| 12 | ADLs: Incontinence | Incontinent = 1, Occasional accidents = 0.5, Without assistance = 0 |
| 13 | ADLs: Needs assistance eating | Need feeding = 1, With some help = 0.5, Without assistance = 0 |
| 14 | IADLs: able to visit neighbors by oneself | Unable to do so = 1, A little difficult = 0.5, Yes = 0 |
| 15 | IADLs: able to shop by oneself if necessary | Unable to do so = 1, A little difficult = 0.5, Yes = 0 |
| 16 | IADLs: able to cook meals by oneself if necessary | Unable to do so = 1, A little difficult = 0.5, Yes = 0 |
| 17 | IADLs: able to wash clothing by oneself | Unable to do so = 1, A little difficult = 0.5, Yes = 0 |
| 18 | IADLs: able to walk continuously for 1 kilometer | Unable to do so = 1, A little difficult = 0.5, Yes = 0 |
| 19 | IADLs: able to lift a weight of 5 kg (such as a heavy bag of groceries) | Unable to do so = 1, A little difficult = 0.5, Yes = 0 |
| 20 | IADLs: able to continuously crouch and stand up three times | Unable to do so = 1, A little difficult = 0.5, Yes = 0 |
| 21 | IADLs: able to use public transportation | Unable to do so = 1, A little difficult = 0.5, Yes = 0 |
| 22 | Able to use chopsticks to eat | No = 1, Yes = 0 |
| 23 | Able to put a hand behind the neck | Neither hand = 1, Right or left hand only = 0.5, Both hands = 0 |
| 24 | Able to put a hand behind the lower back | Neither hand = 1, Right or left hand only = 0.5, Both hands = 0 |
| 25 | Able to raise arm upright | Neither hand = 1, Right or left hand only = 0.5, Both hands = 0 |
| 26 | Able to stand up from sitting in a chair | No = 1, Yes, using hands = 0.5, Yes, without using hands = 0 |
| 27 | Able to pick up a book from the floor | No = 1, Yes, sitting = 0.5, Yes, standing = 0 |
| 28 | Vision loss | Can't see or blind = 1, Can see only = 0.5, Can see and distinguish = 0 |
| 29 | Hearing loss | Yes = 1, No = 0 |
| 30 | Number of serious illnesses in the past 2 years | Two or more illnesses or bedridden = 2, one illness = 1, No = 0 |
| 31 | Suffering from hypertension | Yes = 1, No = 0 |
| 32 | Suffering from diabetes | Yes = 1, No = 0 |
| 33 | Suffering from heart disease | Yes = 1, No = 0 |
| 34 | Suffering from stroke/cerebrovascular disease | Yes = 1, No = 0 |
| 35 | Suffering from bronchitis, emphysema, asthma, or pneumonia | Yes = 1, No = 0 |
| 36 | Suffering from tuberculosis | Yes = 1, No = 0 |
| 37 | Suffering from cancer | Yes = 1, No = 0 |
| 38 | Suffering from arthritis | Yes = 1, No = 0 |
| 39 | Poor interviewer-rated health | Yes = 1, No = 0 |

*ADLs* activities of daily, *IADLs* instrumental activities of daily living.

**Supplementary Table 2**

The numbers (percentage) of the missing variables

| Characteristics ^a^ | Number (%) with missing data |
| --- | --- |
| Marital status | 15 (0.3) |
| Education | 13 (0.3) |
| Smoking status | 4 (0.1) |
| Drinking status | 17 (0.4) |
| Regular exercise | 24 (0.5) |
| Living arrangement | 34 (0.7) |
| Economic status | 8 (0.2) |
| Natural tooth | 55 (1.1) |
| Denture status | 21 (0.4) |
| Body mass index | 50 (1.0) |
| Frequency of fruit intake | 3 (0.1) |
| Frequency of vegetable intake | 1 (0.02) |
| Frequency of meat intake | 6 (0.1) |
| Frequency of fish intake | 4 (0.1) |

*Notes:* ^a^ List only the variables with missing data.

**Supplementary Table 3**

Baseline characteristics of participants who switched cooking oil types during follow-up

| Characteristics | All participants  (n = 4804) | Always vegetable oil  (n = 3933) | Vegetable oil  to animal fat oil  (n = 176) | Always animal fat oil  (n=483) | Animal fat oil  to vegetable oil  (n=212) | P value |
| --- | --- | --- | --- | --- | --- | --- |
| Age (year), mean (SD) | 80.76 (9.58) | 80.38 (9.53) | 82.17 (9.93) | 82.22 (9.59) | 83.37 (9.49) | <0.001 |
| Female, no. (%) | 2392 (49.8) | 1947 (49.5) | 90 (51.1) | 256 (53.0) | 99 (46.7) | 0.382 |
| Urban area, no. (%) | 2226 (46.3) | 1952 (49.6) | 74 (42.0) | 109 (22.6) | 91 (42.9) | <0.001 |
| Married, no. (%) | 2423 (50.4) | 2030 (51.6) | 81 (46.0) | 216 (44.7) | 96 (45.3) | 0.007 |
| Living with family, no. (%) | 3805 (79.2) | 3137 (79.8) | 136 (77.3) | 375 (77.6) | 157 (74.1) | 0.153 |
| Education (year), no. (%) | | | | | | 0.001 |
| 0 | 2425 (50.5) | 1943 (49.4) | 90 (51.1) | 274 (56.7) | 118 (55.7) |  |
| 1-6 | 1739 (36.2) | 1431 (36.4) | 63 (35.8) | 173 (35.8) | 72 (34.0) |  |
| >6 | 640 (13.3) | 559 (14.2) | 23 (13.1) | 36 (7.5) | 22 (10.4) |  |
| Economic independence, no. (%) | 1659 (34.5) | 1492 (37.9) | 38 (21.6) | 86 (17.8) | 43 (20.3) | <0.001 |
| Smoking status, no. (%) | | | | | | 0.005 |
| Never | 3000 (62.4) | 2414 (61.4) | 122 (69.3) | 330 (68.3) | 134 (63.2) |  |
| Current | 1053 (21.9) | 869 (22.1) | 32 (18.2) | 103 (21.3) | 49 (23.1) |  |
| Former | 751 (15.6) | 650 (16.5) | 22 (12.5) | 50 (10.4) | 29 (13.7) |  |
| Drinking status, no. (%) | | | | | | 0.933 |
| Never | 3130 (65.2) | 2571 (65.4) | 118 (67.0) | 310 (64.2) | 131 (61.8) |  |
| Current | 1001 (20.8) | 811 (20.6) | 36 (20.5) | 105 (21.7) | 49 (23.1) |  |
| Former | 673 (14.0) | 551 (14.0) | 22 (12.5) | 68 (14.1) | 32 (15.1) |  |
| Regular exercise, no. (%) | | | | | | <0.001 |
| Never | 2379 (49.5) | 1887 (48.0) | 95 (54.0) | 298 (61.7) | 99 (46.7) |  |
| Current | 2033 (42.3) | 1726 (43.9) | 66 (37.5) | 150 (31.1) | 91 (42.9) |  |
| Former | 392 (8.2) | 320 (8.1) | 15 (8.5) | 35 (7.2) | 22 (10.4) |  |
| Frailty status |  |  |  |  |  | 0.263 |
| Non-frailty | 3738 (77.8) | 3041 (77.3) | 136 (77.3) | 390 (80.7) | 171 (80.7) |  |
| Pre-frailty | 1066 (22.2) | 892 (22.7) | 40 (22.7) | 93 (19.3) | 41 (19.3) |  |
| BMI (kg/m^2^), no. (%) | | | | | | <0.001 |
| Underweight (<18.5) | 2723 (56.7) | 2242 (57.0) | 98 (55.7) | 266 (55.1) | 117 (55.2) |  |
| Normal (18.5-24) | 910 (18.9) | 639 (16.2) | 51 (29.0) | 165 (34.2) | 55 (25.9) |  |
| Overweight (24-28) | 893 (18.6) | 800 (20.3) | 22 (12.5) | 46 (9.5) | 25 (11.8) |  |
| Obese (≥28) | 278 (5.8) | 252 (6.4) | 5 (2.8) | 6 (1.2) | 15 (7.1) |  |
| Natural tooth number, no. (%) | | | | | | 0.001 |
| 20+ | 1391 (29.0) | 1194 (30.4) | 39 (22.2) | 106 (21.9) | 52 (24.5) |  |
| 10-19 | 898 (18.7) | 744 (18.9) | 33 (18.8) | 87 (18.0) | 34 (16.0) |  |
| 1-9 | 1278 (26.6) | 1018 (25.9) | 51 (29.0) | 145 (30.0) | 64 (30.2) |  |
| 0 | 1237 (25.7) | 977 (24.8) | 53 (30.1) | 145 (30.0) | 62 (29.2) |  |
| Denture use, no. (%) | 1835 (38.2) | 1545 (39.3) | 78 (44.3) | 139 (28.8) | 73 (34.4) | <0.001 |
| Frequency of fruit intake, no. (%) | | | | | | <0.001 |
| Daily | 653 (13.6) | 590 (15.0) | 24 (13.6) | 20 (4.1) | 19 (9.0) |  |
| Quite often | 1213 (25.2) | 1046 (26.6) | 42 (23.9) | 87 (18.0) | 38 (17.9) |  |
| Occasionally | 1729 (36.0) | 1377 (35.0) | 53 (30.1) | 218 (45.1) | 81 (38.2) |  |
| Rarely or none | 1209 (25.2) | 920 (23.4) | 57 (32.4) | 158 (32.7) | 74 (34.9) |  |
| Frequency of vegetable intake, no. (%) | | | | | | <0.001 |
| Daily | 2940 (61.2) | 2442 (62.1) | 111 (63.1) | 259 (53.6) | 128 (60.4) |  |
| Quite often | 1473 (30.7) | 1215 (30.9) | 45 (25.6) | 159 (32.9) | 54 (25.5) |  |
| Occasionally | 294 (6.1) | 218 (5.5) | 18 (10.2) | 40 (8.3) | 18 (8.5) |  |
| Rarely or none | 97 (2.0) | 58 (1.5) | 2 (1.1) | 25 (5.2) | 12 (5.7) |  |
| Frequency of meat intake, no. (%) | | | | | | <0.001 |
| Daily | 1503 (31.3) | 1120 (28.5) | 74 (42.0) | 229 (47.4) | 80 (37.7) |  |
| Weekly | 2081 (43.3) | 1753 (44.6) | 68 (38.6) | 185 (38.3) | 75 (35.4) |  |
| Monthly | 572 (11.9) | 484 (12.3) | 19 (10.8) | 37 (7.7) | 32 (15.1) |  |
| Occasionally | 316 (6.6) | 280 (7.1) | 6 (3.4) | 20 (4.1) | 10 (4.7) |  |
| Rarely or none | 332 (6.9) | 296 (7.5) | 9 (5.1) | 12 (2.5) | 15 (7.1) |  |
| Frequency of fish intake, no. (%) | | | | | | <0.001 |
| Daily | 375 (7.8) | 330 (8.4) | 14 (8.0) | 14 (2.9) | 17 (8.0) |  |
| Weekly | 1809 (37.7) | 1564 (39.8) | 53 (30.1) | 151 (31.3) | 41 (19.3) |  |
| Monthly | 1058 (22.0) | 838 (21.3) | 38 (21.6) | 131 (27.1) | 51 (24.1) |  |
| Occasionally | 673 (14.0) | 535 (13.6) | 22 (12.5) | 72 (14.9) | 44 (20.8) |  |
| Rarely or none | 889 (18.5) | 666 (16.9) | 49 (27.8) | 115 (23.8) | 59 (27.8) |  |

*SD* standard deviation; *BMI* Body mass index.

Notes: Values are presented as number (%) or mean ± SD. Differences in characteristics were compared using the χ^2^ test for categorical variables and the t-test for continuous variables.

**Supplementary Table 4**

Demographic and clinical characteristics of the study population after propensity score matching

| Characteristics | All participants  (n = 1350) | Vegetable oil  (n = 675) | Animal fat oil  (n = 675) | *P* value ^a^ |
| --- | --- | --- | --- | --- |
| Age (year), mean (SD) | 82.49 (9.67) | 82.69 (9.89) | 82.29 (9.45) | 0.45 |
| Female, no. (%) | 670 (49.6) | 328 (48.6) | 342 (50.7) | 0.48 |
| Urban area, no. (%) | 391 (29.0) | 190 (28.1) | 201 (29.8) | 0.55 |
| Married, no. (%) | 616 (45.6) | 309 (45.8) | 307 (45.5) | 0.96 |
| Living with family, no. (%) | 1036 (76.7) | 516 (76.4) | 520 (77.0) | 0.85 |
| Education (year), no. (%) | | | | 0.82 |
| 0 | 763 (56.5) | 386 (57.2) | 377 (55.9) |  |
| 1-6 | 467 (34.6) | 228 (33.8) | 239 (35.4) |  |
| ≥6 | 120 (8.9) | 61 (9.0) | 59 (8.7) |  |
| Economic independence, no. (%) | 252 (18.7) | 125 (18.5) | 127 (18.8) | 0.94 |
| Smoking status, no. (%) | | | | 0.94 |
| Never | 891 (66.0) | 444 (65.8) | 447 (66.2) |  |
| Current | 303 (22.4) | 151 (22.4) | 152 (22.5) |  |
| Former | 156 (11.6) | 80 (11.9) | 76 (11.3) |  |
| Drinking status, no. (%) | | | | 0.59 |
| Never | 840 (62.2) | 411 (60.9) | 429 (63.6) |  |
| Current | 311 (23.0) | 160 (23.7) | 151 (22.4) |  |
| Former | 199 (14.7) | 104 (15.4) | 95 (14.1) |  |
| Regular exercise, no. (%) | | | | 0.89 |
| Never | 755 (55.9) | 373 (55.3) | 382 (56.6) |  |
| Current | 483 (35.8) | 245 (36.3) | 238 (35.3) |  |
| Former | 112 (8.3) | 57 (8.4) | 55 (8.1) |  |
| Frailty status | | | | 0.54 |
| Non-frailty | 1076 (79.7) | 533 (79.0) | 543 (80.4) |  |
| Pre-frailty | 274 (20.3) | 142 (21.0) | 132 (19.6) |  |
| BMI (kg/m^2^), no. (%) | | | | 0.90 |
| Underweight (<18.5) | 762 (56.4) | 383 (56.7) | 379 (56.1) |  |
| Normal (18.5-24) | 404 (29.9) | 200 (29.6) | 204 (30.2) |  |
| Overweight (24-28) | 146 (10.8) | 75 (11.1) | 71 (10.5) |  |
| Obese (≥28) | 38 (2.8) | 17 (2.5) | 21 (3.1) |  |
| Natural tooth number, no. (%) | | | | 0.99 |
| 20+ | 316 (23.4) | 159 (23.6) | 157 (23.3) |  |
| 10-19 | 240 (17.8) | 122 (18.1) | 118 (17.5) |  |
| 1-9 | 394 (29.2) | 195 (28.9) | 199 (29.5) |  |
| 0 | 400 (29.6) | 199 (29.5) | 201 (29.8) |  |
| Denture use, no. (%) | 412 (30.5) | 203 (30.1) | 209 (31.0) | 0.77 |
| Frequency of fruit intake, no. (%) | | | | 0.80 |
| Daily | 74 (5.5) | 35 (5.2) | 39 (5.8) |  |
| Quite often | 237 (17.6) | 113 (16.7) | 124 (18.4) |  |
| Occasionally | 598 (44.3) | 301 (44.6) | 297 (44.0) |  |
| Rarely or none | 441 (32.7) | 226 (33.5) | 215 (31.9) |  |
| Frequency of vegetable intake, no. (%) | | | | 0.96 |
| Daily | 762 (56.4) | 380 (56.3) | 382 (56.6) |  |
| Quite often | 416 (30.8) | 206 (30.5) | 210 (31.1) |  |
| Occasionally | 115 (8.5) | 60 (8.9) | 55 (8.1) |  |
| Rarely or none | 57 (4.2) | 29 (4.3) | 28 (4.1) |  |
| Frequency of meat intake, no. (%) | | | | 0.81 |
| Daily | 558 (41.3) | 269 (39.9) | 289 (42.8) |  |
| Weekly | 537 (39.8) | 277 (41.0) | 260 (38.5) |  |
| Monthly | 142 (10.5) | 73 (10.8) | 69 (10.2) |  |
| Occasionally | 62 (4.6) | 32 (4.7) | 30 (4.4) |  |
| Rarely or none | 51 (3.8) | 24 (3.6) | 27 (4.0) |  |
| Frequency of fish intake, no. (%) | | | | 0.96 |
| Daily | 68 (5.0) | 37 (5.5) | 31 (4.6) |  |
| Weekly | 380 (28.1) | 189 (28.0) | 191 (28.3) |  |
| Monthly | 347 (25.7) | 171 (25.3) | 176 (26.1) |  |
| Occasionally | 230 (17.0) | 116 (17.2) | 114 (16.9) |  |
| Rarely or none | 325 (24.1) | 162 (24.0) | 163 (24.1) |  |

*SD* standard deviation; *BMI* Body mass index.

Notes: Values are presented as number (%) or mean ± SD. ^a^ Differences in characteristics were compared using the χ^2^ test for categorical variables and the t-test for continuous variables.

**Supplementary Table 5**

Hazard ratios for incident frailty according to cooking oil categories estimated by competing risk model

| Cooking oil | Unadjusted Model | Model 1 | Model 2 |
| --- | --- | --- | --- |
|  | HR (95% CI) | HR (95% CI) | HR (95% CI) |
| Vegetable oil | Reference | Reference | Reference |
| Animal fat oil | 0.85 (0.73-0.99) | 0.79 (0.68-0.92) | 0.83 (0.71-0.98) |

*HR* hazard ratio; *CI* confidence interval.

Notes: Model 1: adjusted for age and sex.

Model 2: further adjusted for residence, education, living arrangement, economic status, marital status, smoking status, drinking status, regular exercise, the number of natural teeth, denture use, body mass index, baseline frailty status, frequency of fruit intake, frequency of vegetable intake, frequency of meat intake, and frequency of fish intake.

**Supplementary Table 6**

Sensitive analysis of the association between cooking oil and incident frailty

| Variables | Unadjusted model  HR (95% CI) | Model 1  HR (95% CI) | Model 2  HR (95% CI) |
| --- | --- | --- | --- |
| After propensity score matching (n = 1336) | | | |
| Vegetable oil | Reference | Reference | Reference |
| Animal fat oil | 0.68 (0.56-0.83) | 0.68 (0.56-0.83) | 0.70 (0.57-0.86) |
| Complete cases (n = 4576) | | | |
| Vegetable oil | Reference | Reference | Reference |
| Animal fat oil | 0.85 (0.73-0.99) | 0.71 (0.61-0.83) | 0.74 (0.63-0.88) |
| Excluding frailty that occurred during the second year of follow-up (n = 4473) | | | |
| Vegetable oil | Reference | Reference | Reference |
| Animal fat oil | 0.84 (0.72-0.99) | 0.69 (0.59-0.82) | 0.74 (0.63-0.88) |
| With extended adjustments ^a^ (n = 4838) | | | |
| Vegetable oil | Reference | Reference | Reference |
| Animal fat oil | 0.84 (0.72-0.98) | 0.69 (0.59-0.80) | 0.77 (0.65-0.91) |
| Competing risk model with extended adjustments ^a^ (n = 6029) | | | |
| Vegetable oil | Reference | Reference | Reference |
| Animal fat oil | 0.85 (0.73-0.99) | 0.79 (0.68-0.92) | 0.86 (0.73-0.99) |

*HR* hazard ratio; *CI* confidence interval.

Notes: ^a^ Additionally adjusted for sleep time, sleep quality, frequency of egg intake, frequency of bean intake,

frequency of milk intake, and amount of staple food per day.

Model 1: adjusted for age and sex.

Model 2: further adjusted for residence, education, economic status, marital status, living arrangement, smoking status, drinking status, regular exercise, sleep time, the number of natural teeth, denture use, body mass index, frailty status, frequency of fruit intake, frequency of vegetable intake, frequency of meat intake, and frequency of fish intake.


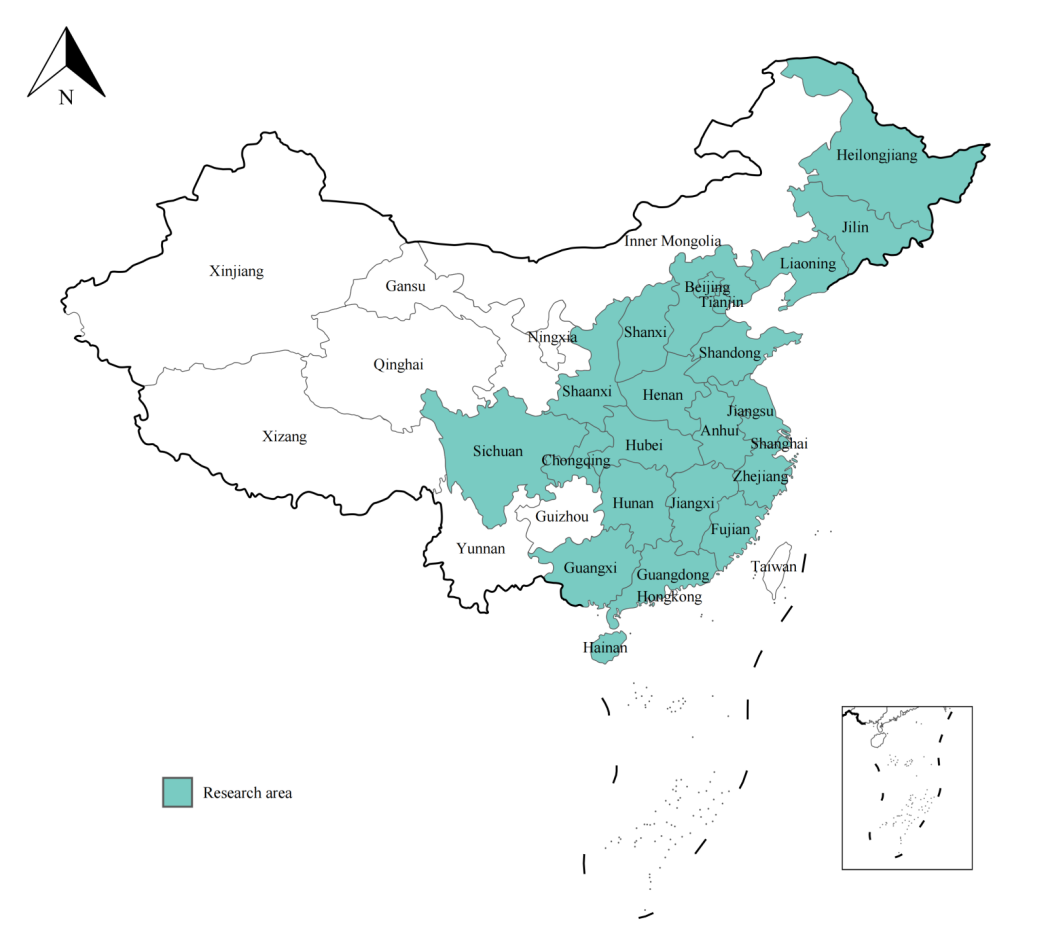
**Supplementary Figure 1** The 23 research areas included in the Chinese Longitudinal Healthy Longevity Survey (CLHLS) in mainland China in the 2011/2012 wave


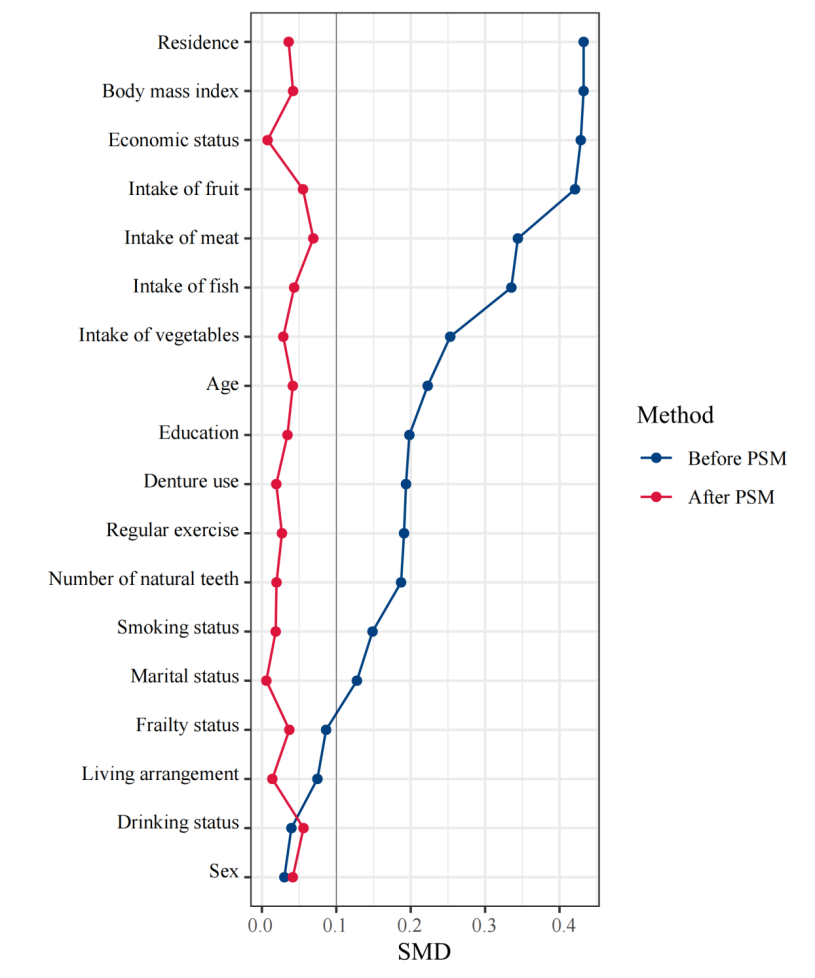


**Supplementary Figure 2** The standardized mean differences (SMD) of the variables after propensity score matching (PSM).
